# Phase Ib/II study on the safety, tolerability, and preliminary efficacy of pegylated irinotecan (JK1201I) as second‐line monotherapy for patients with small‐cell lung cancer

**DOI:** 10.1002/cam4.70059

**Published:** 2024-09-03

**Authors:** Jieran Long, Xuefei Li, Lin Wu, Guohua Yu, Aimin Zang, Yanqiu Zhao, Jinsheng Shi, Ligong Nie, Xuan Zhao, Jian Fang

**Affiliations:** ^1^ Department of Thoracic Oncology Beijing University Cancer Hospital Beijing China; ^2^ Department of Clinical Medicine and Pharmacology JenKemTechnology Co., Ltd. (Tian Jin) Tianjin China; ^3^ Thoracic Medicine Department Hunan Cancer Hospital Changsha Hunan China; ^4^ Medicine Oncology Department Weifang People's Hospital Weifang Shandong China; ^5^ Internal Medicine Oncology Ward Affiliated Hospital of Hebei University Baoding Hebei China; ^6^ Department of Respiratory Medicine Henan Cancer Hospital Zhengzhou Henan China; ^7^ Internal Medicine Oncology Ward Cangzhou People's Hospital Cangzhou Hebei China; ^8^ Pulmonary and Critical Care Medicine Department, Beijing University First Hospital Beijing China; ^9^ JenKem Technology Co., Ltd. (Tian Jin) Tianjin China

**Keywords:** overall survival, safety, second‐line monotherapy, small‐cell lung cancer

## Abstract

**Purpose:**

To evaluate the safety, tolerability, and preliminary efficacy of multiple doses of pegylated irinotecan (JK1201I) as a second‐line monotherapy for treating small‐cell lung cancer (SCLC) patients.

**Methods:**

According to the “3 + 3” dose‐escalation principle, patients received intravenous JK1201I at 180 or 220 mg/m^2^ once every 3 weeks for four cycles. Progression‐free survival (PFS), overall survival (OS), median progression‐free survival (mPFS), and median overall survival (mOS) were evaluated. The Kaplan–Meier method was used to analyze PFS and overall OS. Brookmeyer and Crowley's method was used for mPFS and mOS.

**Results:**

This study included 29 patients with stage III–IV SCLC (stage IIIa, *n* = 1; stage IIIb, *n* = 1; and stage IV, *n* = 27). Of these, 26 patients were enrolled in the 180 mg/m^2^ dose group, and 3 patients were enrolled in the 220 mg/m^2^ dose group. No dose‐limiting toxicity (DLT) was noted during the first 28 days of treatment. Grade 3 or higher adverse events were recorded in the 180 mg/m^2^ group, including diarrhea (11.5%, 3/26), neutropenia (7.7%, 2/26), and leukopenia (7.7%, 2/26). In the 220 mg/m^2^ group, one patient (33.3%, 1/3) experienced neutropenia or leukopenia. In the 180 mg/m^2^ group, 38.5% (10/26) of patients achieved an objective response rate (ORR), with a disease control rate (DCR) of 73.1% (19/26). The mPFS and mOS were 3.4 and 12.1 months, respectively. In the 220 mg/m^2^ group, one patient had stable disease, and one had progressive disease (PD). The ORR, DCR, mPFS, and mOS were 0% (0/3) and 33.3% (1/3), 2.7 months and 2.7 months, respectively.

**Conclusion:**

JK1201I exhibits promising efficacy and relatively low toxicities as a second‐line monotherapy for SCLC, warranting further large‐scale clinical studies to evaluate its efficacy in greater detail.

## 1 INTRODUCTION

Small‐cell lung cancer (SCLC) occupies 15%–17% of total lung cancer diagnoses.^1^ The mOS of SCLC patients is approximately 10 months from the time of diagnosis in 60%–70% of patients with extensive stage (ES) or stage IV SCLC. Chemotherapy and radiotherapy increase survival by 8 months in patients with M1b disease and by 12 months in patients with M1a disease.^2^ SCLC is sensitive to platinum‐based chemotherapy. These patients underwent platinum‐based chemotherapy and reported an ORR of 41.3% (95% confidence interval [CI]: 25.5–59.3) and an mOS of 10.4 months (95% CI: 8.1–14). Thus, topotecan was approved by the US Food and Drug Administration (FDA) and the European Medicines Agency.^3^ Nevertheless, it is not widely used with hematological toxicities.^4^, ^5^

Lurbinectedin was approved by the FDA in June 2020 as second‐line chemotherapy for SCLC patients undergoing progression or after platinum‐based therapy. In a prospective, single‐arm phase II study, Trigo et al.^6^ reported an ORR of 35.2% and an OS of 9.3 months with lurbinectedin treatment. In another study, Paz‐Ares et al.^7^ explored liposomal irinotecan (Onivyde) as a second‐line monotherapy in SCLC patients. The primary endpoint was not achieved because the mOS with Onivyde was 7.9 (95% CI: 6.9–9.2) months compared with 8.3 (95% CI: 7.3–9.1) months with topotecan in a phase III study. However, the efficacy of this drug in SCLC appears to be inconsistent. In another study, a doubling of ORR was observed (44.1% vs. 21.6%).^7^, ^8^, ^9^

JK1201I is a novel trivalent pegylated irinotecan containing a 20 kDa PEG chain, a biodegradable oligo‐peptidyl linker, and three irinotecan hydrochloride bonds, demonstrating good tolerability and potential efficacy.^10^, ^11^ In a phase I trial (NCT04366648), no serious adverse event (SAE) of grade 3 or above was in 19 patients with advanced solid tumors administrated six doses of JK1201I (50, 75, 100, 125, 150, and 180 mg/m^2^). A complete response was not found. However, one patient with SCLC in the 150‐mg/m^2^ dose group exhibited a partial response (PR). Here, we reported the final analysis of the phase II study on the safety, tolerability, and preliminary efficacy of JK1201I as second‐line chemotherapy.

### 1.1 Study design

This present study is a single‐arm, open‐label, multicenter, phase Ib/II study (ClinicalTrials.gov identifier: NCT05158491). The safety of JK1201I was evaluated in the dose‐exploration phase with doses ranging from 180 to 220, 260, and 300 mg/m^2^ using a “3 + 3” design. The dose‐expansion phase was conducted with 20 patients in each dose group.

Adult SCLC patients aged 18–70 years were included. The patients were enrolled from seven hospitals between December 4, 2021, and October 18, 2022 (Table 1). The inclusion criteria were as follows: (1) an Eastern Cooperative Oncology Group performance status (ECOG‐PS) score of 0 or 1; (2) disease progression confirmed by radiology during or after first‐line platinum‐based chemotherapy for 6 months or less; (3)at least one assessable lesion using the Response Evaluation Criteria in Solid Tumors (RECIST; version 1.1)^12^; (4) an expected survival time of at least 12 weeks; (5) favorable organ and hematopoietic function and no abnormalities in heart, lung, liver, or kidney function or immune deficiency: White blood cell count of ≥3.5 × 10^9^/L; Absolute neutrophil count (ANC) of ≥1.5 × 10^9^/L; Hemoglobin levels of ≥90 g/L (without recent blood transfusion within 2 weeks); Platelet count of ≥100 × 10^9^/L; Alanine transaminase (ALT) and aspartate aminotransferase (AST) levels within ≤2.5 times the upper limit of normal (ULN), and in cases of liver metastases, AST and ALT levels within ≤5 times the ULN; Serum creatinine concentration within ≤1.5 times the ULN or a creatinine clearance rate of >50 mL/min; Total bilirubin concentration within ≤1.5 times the ULN; Activated partial thromboplastin time within ≤1.5 times the ULN and international normalized ratio or prothrombin time within ≤1.5 times the ULN (without anticoagulant therapy); (6) Male and female patients of reproductive age willing to use effective non‐drug contraceptives from the day of signing the informed consent form until 6 months after the last treatment; (7) Female patients with a negative blood‐based pregnancy test result within 7 days before the first treatment; and (8) Those providing informed consent.

**TABLE 1:** Hospital list and enrolled number of patients.

No.	Name of the hospital	Department	Enrolled number of patients
1	Peking University Cancer Hospital	Thoracic Oncology	14
2	Hunan Cancer Hospital	Thoracic Medicine Department II	4
3	Weifang People's Hospital	Medicine Oncology	3
4	Affiliated Hospital of HEBEI University	Internal Medicine Oncology ward	3
5	Henan Cancer Hospital	Respiratory medicine	2
6	Cangzhou People's Hospital	Internal Medicine Oncology ward	2
7	Peking University First Hospital	Pulmonary and Critical Care Medicine	1
Total			29

The exclusion criteria were as follows: (1)Allergy history or sensitivity to JK1201I or any of its components; (2) treated with topoisomerase I inhibitors, or had undergone chemotherapy or immunotherapy in the last 4 weeks, or treatment with nitrosourea or mitomycin C in the last 6 weeks; (3) palliative radiotherapy or biotherapy in the last 2 weeks, or hormonotherapy involving oral prednisone (over 50 mg) in the past week; (4) took a strong CYP3A4 inducer in the past 2 weeks, or strong inhibitors of CYP3A4 or UGT1A1 in the past week; (5) experienced chronic cardiovascular diseases, including cardiac dysfunction of grade 2 or higher (following the New York Heart Association standards) and pericardial effusion, within the past 6 months; (6) underwent symptomatic brain metastases or central nervous system metastases; (7) received full brain radiotherapy for metastases lasting week or stereotactic body radiation therapy lasting less than 3 days; and (8) pregnant individuals, those with severe infectious diseases, and other severe complications.

## 2 METHODS

### 2.1 Treatment

JK1201I is available as a 40 mg/vial (lyophilized powder; equivalent to irinotecan hydrochloride) and is manufactured by Beijing SL Co., Ltd. (commissioned by JenKem Technology Co., Ltd., Tian Jin; Lot. 20,200,902 and 20,210,402). The drug was stored at 2–8°C. The powdered form was diluted in 500 mL of 0.9% sodium chloride solution. Patients received intravenous JK1201I for 240 ± 15 min once every 3 weeks at a dose of 180 or 220 mg/m^2^ (as irinotecan hydrochloride) for four cycles. Survival data were collected every 3 months until the last follow‐up with the patients or at the end of the study.

Blood samples for pharmacokinetics analysis were collected 60 min before drug administration: 10 min ± 5 min after the start of infusion; 10 min ± 5 min before the end of infusion; and 0 min ± 5 min, 20 min ±5 min, 1 h ± 5 min, 3 h ± 15 min, 6 h ± 30 min, 12 h ± 30 min, 24 h ± 1 h, 48 h ± 2 h, 72 h ± 2 h, 168 h ± 2 h, 240 h ± 2 h, and 336 h ± 4 h after the end of infusion in the first two treatment cycles during the dose‐exploration from 4 in the 180‐mg/m^2^ group and 3 in the 220‐mg/m^2^ group.

Target lesions were measured at baseline using computed tomography (CT) or magnetic resonance imaging (MRI) within 4 weeks before drug administration. After drug administration, lesions were measured at the end of cycles 2 and 4 or before withdrawal.

### 2.2 Baseline characteristics

Data on patients' age, sex, height, body weight, body mass index (BMI), ECOG‐PS score, tumor stage, time since diagnosis, baseline metastasis, and previous therapies were collected. Table 2 lists the baseline characteristics. The baseline of the lesions was determined based on CT/MRI results obtained within 4 weeks prior to the first administration. Following the initiation of treatment, patients were evaluated using CT/MRI at the end of the second and fourth cycles or prior to withdrawal.

**TABLE 2:** Demographics and disease characteristics of all patients at baseline (FAS).

Characteristics	180 mg/m^2^	220 mg/m^2^	Total
*N*	26	3	29
Age (y)			
Mean ± SD	58.7 ± 8.64	60.3 ± 12.42	58.9 ± 8.83
Median (range)	61.5 (35–68)	67.0 (46–68)	62.0 (35–68)
Sex no. (%)			
Male	23 (88.5)	3 (100.0)	26 (89.7)
Female	3 (11.5)	0 (0)	3 (10.3)
Height (cm)			
Mean ± SD	167.94 ± 6.035	173.33 ± 5.132	168.50 ± 6.099
Median (range)	169.00 (156.4–178)	172.00 (169–179)	170.00 (156.4–179)
Bodyweight (kg)			
Mean ± SD	72.45 ± 13.080	73.00 ± 14.933	72.50 ± 12.989
Median (range)	68.50 (47.7–118)	67.00 (62–90)	68.00 (47.7–118)
BMI (kg/m^2^)			
Mean ± SD	25.613 ± 3.808	24.148 ± 3.445	25.461 ± 3.742
Median (range)	25.357 (19.50–37.24)	22.647 (21.71–28.09)	25.059 (19.50–37.24)
ECOG‐PS score, no. (%)			
0 point, *n* (%)	9 (34.6)	1 (33.3)	10 (34.5)
1 point, *n* (%)	17 (65.4)	2 (66.7)	19 (65.5)
Diagnosis, no. (%)			
SCLC	26 (100.0)	3 (100.0)	29 (100.0)
Time since diagnosis (months)			
Mean ± SD	10.13 ± 7.610	26.67 ± 18.698	11.84 ± 10.147
Median (range)	7.8 (3.9–41.8)	24.8 (9.0–46.2)	8.77 (3.9–46.2)
Disease status, no. (%)			
IIIa	1 (3.8)	0 (0)	1 (3.4)
IIIb	1 (3.8)	0 (0)	1 (3.4)
IV	24 (92.3)	3 (100.0)	27 (93.1)
Metastatic, no. (%)			
M0	2 (7.7)	0 (0)	2 (6.9)
M1	24 (92.3)	3 (100.0)	27 (93.1)
Key metastatic site(s), no. (%)			
Brain	13 (50.0)	0 (0)	13 (44.8)
Hepatic	10 (38.5)	2 (66.7)	12 (41.4)
Lymphatic	22 (84.6)	3 (100.0)	25 (86.2)
Bone	13 (50.0)	0 (0)	13 (44.8)
Previous therapies, no. (%)			
Platinum‐based	26 (100.0)	3 (100.0)	29 (100.0)
Immunotherapy	18 (69.2)	3 (100.0)	21 (72.4)
Radiotherapy	15 (57.7)	3 (100.0)	18 (62.1)
The best response to previous therapies, no. (%)			
Complete response	0 (0.0)	0 (0.0)	0 (0.0)
Partial response	12 (46.2)	3 (100.0)	15 (51.7)
Stable disease	2 (7.7)	0 (0.0)	2 (6.9)
Progressive disease	9 (34.6)	0 (0.0)	9 (31.0)
Unknown	3 (11.5)	0 (0.0)	3 (10.3)

Abbreviations: BMI, body mass index; ECOG‐PS, Eastern Cooperative Oncology Group performance status; FAS, full analysis set; SCLC, small‐cell lung cancer; SD, standard deviation;

### 2.3 Assessments and end points

Dose‐limiting toxicities (DLTs) were categorized in the initial three patients in both the 180‐ and 220‐mg/m^2^ groups during the first 21 days. DLTs were evaluated in four patients in the 180‐mg/m^2^ group and three in the 220‐mg/m^2^ group. The AEs^1^: grade 4 neutropenia persisting for at least 3 days or grade 3 febrile neutropenia (ANC of <1000/mm^3^ with an oral temperature over 38.3°C measured once or ≥38.0°C lasting 1 h)^2^; grade 3 thrombocytopenia (25 × 10^9^/L ≤ normal platelet count ≤50 × 10^9^/L) with bleeding, or grade 4 thrombocytopenia with or without bleeding^3^; any grade 4 hematologic toxicity^4^; grade 3 or higher non‐hematological toxicities excluding hair loss, fatigue, and nausea, vomiting, and diarrhea without maximum supportive therapy. AEs and treatment‐emergent AEs (TEAEs) were documented 1 month after the final treatment. Severity was graded using the common terminology criteria for adverse events, version 5.0.

Secondary endpoints encompassed the preliminary efficacy of JK1201I as a second‐line monotherapy in SCLC patients and the evaluation of PFS and OS to determine the recommended dose for a phase III study. PFS, as per RECIST (version 1.1), is the duration from the start of chemotherapy to the day of radiographic confirmation of disease progression or death. OS means the period from the commencement of chemotherapy to the date of death from any cause or the last follow‐up.

A post hoc supplementary analysis of efficacy outcomes was conducted in the 180‐mg/m^2^ group, considering factors including age (below or above 65 years), presence or absence of brain/bone/lymphatic metastases at baseline, the interval between the conclusion of first‐line treatment and the initial administration of JK1201I (more or less than 3 months), ECOG scores of 0 or 1 before the first administration of JK1201I, receipt or non‐receipt of immunotherapy as first‐line treatment, exposure time shorter or longer than the median, and the occurrence or non‐occurrence of any TEAEs.

### 2.4 Analysis methods

The Kaplan–Meier (KM) method was employed for PFS and OS. The Brookmeyer and Crowley method was for the mPFS and mOS. The ORR was assessed using the Clopper–Pearson method. Statistical analyses were conducted using SAS software (version 9.4 or higher; Shanghai BioGuider Medical Technology Co., Ltd.).

## 3 RESULTS

### 3.1 Baseline characteristics

A total of 29 SCLC patients were enrolled in this study, with 26 in the 180‐mg/m^2^ group and three in the 220‐mg/m^2^ group. The mean age of patients was 58.9 ± 8.83 (range: 35–68) years. Twenty‐seven patients (93.1%) were diagnosed with stage IV SCLC, one (3.4%) was with stage IIIa SCLC, and one (3.4%) was with stage IIIb SCLC (Table 2). Twenty‐seven patients (93.1%) exhibited metastases at baseline (liver, *n* = 12 [41.4%]; brain, *n* = 13 [44.8%]; bone, *n* = 13 [44.8%]; lymph node, *n* = 25 [86.2%]). All patients (100%) received platinum‐based chemotherapy as their first‐line treatment, 21 (72.4%) received immunotherapy, and 18 (62.1%) received radiotherapy.

Sixteen patients (55.2%) received all four cycles of JK1201I treatment, while 13 patients (44.8%) discontinued their treatment cycles: Six showed progressive disease (PD), two withdrew voluntarily, one developed rapid‐onset anaphylaxis, one died of pulmonary mucormycosis, one experienced treatment delay for over 2 weeks due to anemia, one developed diarrhea and grade 4 neutropenia with UGT1A1*6 (A/A) homozygosity mutations, and one refused to receive follow‐up examinations. All patients were included in the full analysis set (FAS) and safety set (SS), 26 were included in the efficacy analysis set (EAS), seven were included in the pharmacokinetics concentration set (PKCS), and six were included in the pharmacokinetics parameter set (PKPS) and DLT set (DLTS; Table 3). Although DLT was not observed in all dosage settings, dose exploration above 220 mg/m^2^ and dose expansion of over 180 mg/m^2^ were not performed. After discussion among the investigators, the dose of 180 mg/m^2^ of JK1201I was recommended for the phase III study of SCLC patients.

**TABLE 3:** Distribution of patients.

	180 mg/m^2^	220 mg/m^2^	Total
Enrolled	26 (78.8)	3 (9.1)	29 (87.9)
Received treatment	26 (100.0)	3 (100.0)	29 (100.0)
Finished all four treatment cycles	15 (57.7)	1 (33.3)	16 (55.2)
Did not finish four treatment cycles	11 (42.3)	2 (66.7)	13 (44.8)
Progressive disease, *n* (%)	5 (19.2)	1 (33.3)	6 (20.7)
Withdraw voluntarily, *n* (%)	1 (3.8)	1 (33.3)	2 (6.9)
Rapid‐onset anaphylaxis, *n* (%)	1 (3.8)	0 (0)	1 (3.4)
Death, *n* (%)	1 (3.8)	0 (0)	1 (3.4)
Treatment delay for more than 2 weeks, *n* (%)	1 (3.8)	0 (0)	1 (3.4)
SAEs with UGT1A1 × 6 (A/A) homozygosity mutations, *n* (%)	1 (3.8)	0 (0)	1 (3.4)
Refused to visit for the follow‐up examination, *n* (%)	1 (3.8)	0 (0)	1 (3.4)
FAS	26	3	29
EAS	24	2	26
SS	26	3	29
PKCS	4	3	7
PKPS	3	3	6
DLTS	3	3	6

Abbreviations: DLTS, dose‐limited toxicity set; EAS, efficacy analysis set; FAS, full analysis set; PKCS, pharmacokinetics concentration set; PKPS, pharmacokinetics parameter set; SAE, serious adverse event; SS, safety set.

### 3.2 Pharmacokinetics analyses

**FIGURE 1:**
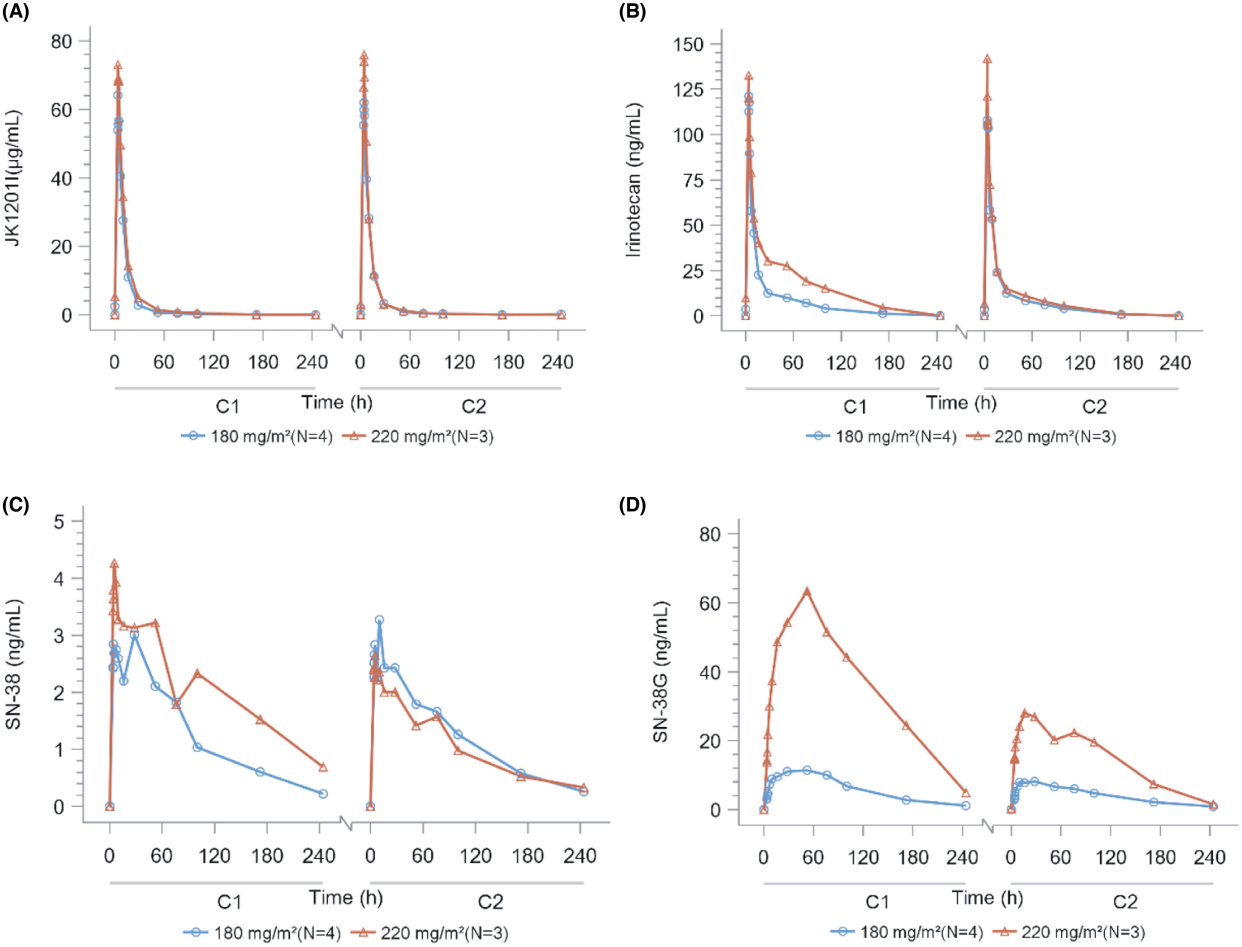
Plasma concentration‐time profiles for JK1201I, free irinotecan, SN‐38, and SN‐38G.

Figure 1 presents plasma concentration‐time profiles for JK1201I, free irinotecan, SN‐38, and SN‐38G. Tables 4, 5, 6 detail the primary PK parameters. At 180 and 220 mg/m^2^ doses, most PK parameters in cycles 1 and 2, including maximum concentration (*C*_max_), area under the curve (0–*t*) (AUC_0–*t*_), and area under the curve (0–∞) (AUC_0–∞_), escalated with dose increases. The inclusion of one patient in the 95% CI range of β indicated linear dynamics between doses and PK parameters. We need information with the slightly broad 95% CI range of β (Table 6). Most PK parameters displayed no significant variation between cycles 1 and 2. The half‐lives of free irinotecan and SN38 derived from JK1201I were 40.75 and 87.57 hours, with C_max_ values of 121.10 and 3.537 ng/mL (Table 4). Compared to data on irinotecan hydrochloride (CPT‐11) from Pitot et al.,^13^ the half‐lives of free irinotecan and SN38 were longer at 12.9 and 27.1 h. The respective C_max_ values were >2810 and 41 ng/mL. JK1201I extends the half‐lives and tumor exposure of both irinotecan and SN‐38, reducing the C_max_ values. The average exposure dose at 180 mg/m^2^ was 984.910 ± 433.417 mg, with an average of 48.7 ± 26.39 days (Table 5).

**TABLE 4:** Pharmacokinetic parameters of each composition in the 180‐ and 220‐mg/m^2^ groups in cycle 1.

	180 mg/m^2^	220 mg/m^2^
JK1201I		
*C*_max_ (μg/mL)	64.10 ± 2.862	73.80 ± 15.752
*T*_max_ ^a^	3.940 ± 0.085	4.180 ± 0.185
*t*_1/2_ (h)	23.614 ± 14.816	37.640 ± 7.520
AUC_0–*t*_ (h*μg/mL)	614.666 ± 73.365	827.246 ± 322.719
AUC_0–∞_ (h*μg/mL)	619.609 ± 74.198	850.303 ± 347.099
Free irinotecan		
*C*_max_ (ng/mL)	121.10 ± 28.054	134.23 ± 32.300
*T*_max_ (h)^a^	3.940 ± 0.085	3.963 ± 0.168
*t*_1/2_ (h)	40.753 ± 6.101	44.155 ± 7.567
AUC_0–*t*_ (h*ng/mL)	1787.448 ± 431.142	3779.802 ± 2778.103
AUC_0–∞_ (h*ng/mL)	1909.396 ± 419.817	4103.051 ± 3168.892
SN38		
*C*_max_ (ng/mL)	3.537 ± 0.183	4.387 ± 2.560
*T*_max_ (h)^a^	13.650 ± 12.018	21.213 ± 27.857
*t*_1/2_ (h)	87.570 ± 17.186	96.125 ± 34.700
AUC_0–*t*_ (h*ng/mL)	287.085 ± 64.380	439.815 ± 313.965
AUC_0–∞_ (h*ng/mL)	339.791 ± 41.490	742.028 ± 744.545
SN‐38G		
*C*_max_ (ng/mL)	13.33 ± 3.873	63.37 ± 50.538
*T*_max_ (h)^a^	31.373 ± 17.543	51.337 ± 1.773
*t*_1/2_ (h)	56.050 ± 6.631	58.961 ± 29.803
AUC_0–*t*_ (h*ng/mL)	1425.999 ± 666.206	12,339.051 ± 14,205.457
AUC_0–∞_ (h*ng/mL)	1527.856 ± 734.000	17,737.766 ± 15,342.630

^a^ Time relative to the start of the infusion.

Abbreviations: AUC_0–∞_, the area under the curve (0–∞); AUC_0–*t*_, the area under the curve (0–*t*); *C*_max_, maximum concentration; *T*_max_, time to maximum concentration;

**TABLE 5:** Analysis of drug exposure (FAS).

Item	180 mg/m^2^	220 mg/m^2^
Drug exposure (mg)		
*N*	26	3
Mean ± SD	984.910 ± 433.417	1106.667 ± 631.672
Median	1209.200	750.000
Q1–Q3	636.000–1317.600	734.000–1836.000
Min–Max	12.46–1728.00	734.00–1836.00
Total infusion duration (h)		
*N*	26	3
Mean ± SD	12.179 ± 5.108	10.067 ± 5.669
Median	15.825	8.333
Q1–Q3	7.867–16.017	5.467–16.400
Min–Max	0.17–16.67	5.47–16.40
Time of drug exposure (day)		
*N*	26	3
Mean ± SD	48.7 ± 26.39	40.3 ± 20.50
Median	63.5	29.0
Q1–Q3	24.0–65.0	28.0–64.0
Min–Max	1.00–82.00	28.00–64.00
Intensity of drug exposure (mg/m^2^/3 weeks)		
*N*	26	3
Mean ± SD	160.636 ± 33.915	194.872 ± 19.111
Median	170.747	184.422
Q1–Q3	158.785–177.398	183.265–216.930
Min–Max	7.15–180.57	183.27–216.93

Abbreviations: Max, maximum; Min, minimum; SD, standard deviation.

**TABLE 6:** Dose proportionality for JK1201I at 180–220 mg/m^2^ (PKPS).

Item	C1			Item	C2		
Parameters	Estimate	SE	95% CI	Parameters	Estimate	SE	95% CI
JK1201I				JK1201I			
*C*_max_ (*n* = 6)				*C*_max_ (*n* = 4)			
*α*	0.9	3.386	−8.501–10.301	*α*	−1.094	1.582	−7.899–5.710
*β*	0.628	0.64	−1.148–2.403	*β*	1.005	0.299	−0.280–2.291
AUC_0–*t*_ (*n* = 6)				AUC_0–*t*_ (*n* = 4)			
*α*	−0.188	5.814	−16.332–15.955	*α*	3.802	2.794	−8.218–15.822
*β*	1.272	1.098	−1.777–4.321	*β*	0.52	0.528	−1.751–2.790
AUC_0–∞_ (*n* = 6)				AUC_0–∞_ (*n* = 4)			
*α*	−0.574	6.05	−17.372–16.224	*α*	4.004	2.605	−7.205–15.213
*β*	1.348	1.143	−1.825–4.521	*β*	0.485	0.492	−1.632–2.602
Irinotecan				Irinotecan			
*C*_max_ (*n* = 6)				*C*_max_ (*n* = 4)			
*α*	2.177	5.413	−12.851–17.205	*α*	0.826	8.134	−34.173–35.825
*β*	0.501	1.022	−2.338–3.339	*β*	0.757	1.536	−5.854–7.368
AUC_0–*t*_ (*n* = 6)				AUC_0–*t*_ (*n* = 4)			
*α*	−8.139	11.223	−39.298–23.021	*α*	2.904	10.132	−40.691–46.499
*β*	3.005	2.12	−2.881–8.891	*β*	0.871	1.914	−7.363–9.106
AUC_0–∞_ (*n* = 6)				AUC_0–∞_ (*n* = 4)			
*α*	−7.933	11.563	−40.037–24.170	*α*	3.894	8.165	−31.237–39.024
*β*	2.979	2.184	−3.085–9.043	*β*	0.704	1.542	−5.932–7.339
SN‐38				SN‐38			
*C*_max_ (*n* = 6)				*C*_max_ (*n* = 4)			
*α*	−1.626	8.345	−24.795–21.542	*α*	4.734	4.102	−12.915–22.383
*β*	0.556	1.576	−3.820–4.932	*β*	−0.685	0.775	−4.019–2.648
AUC_0–*t*_ (*n* = 6)				AUC_0–*t*_ (*n* = 4)			
*α*	−1.206	11.874	−34.174–31.762	*α*	11.411	8.036	−23.163–45.986
*β*	1.319	2.243	−4.909–7.546	*β*	−1.111	1.518	−7.642–5.419
AUC_0–∞_ (*n* = 5)				AUC_0–∞_ (*n* = 4)			
*α*	−5.461	17.525	−61.235–50.312	*α*	7.697	6.259	−19.233–34.627
*β*	2.173	3.323	−8.402–12.748	*β*	−0.381	1.182	−5.467–4.706
SN‐38G				SN‐38G			
*C*_max_ (*n* = 6)				*C*_max_ (*n* = 4)			
*α*	−30.247	16.978	−77.384–16.891	*α*	−24.753	18.047	−102.403–52.897
*β*	6.318	3.207	−2.585–15.222	*β*	5.173	3.409	−9.493–19.840
AUC_0–*t*_ (*n* = 6)				AUC_0–*t*_ (*n* = 4)			
*α*	−35.819	22.328	−97.812–26.173	*α*	−20.206	17.572	−95.810–55.398
*β*	8.282	4.217	−3.427–19.992	*β*	5.213	3.319	−9.067–19.494
AUC_0–∞_ (*n* = 5)				AUC_0–∞_ (*n* = 4)			
*α*	−52.166	16.901	−105.951–1.619	*α*	−21.529	18.81	−102.461–59.403
*β*	11.442	3.204	1.244–21.640	*β*	5.483	3.553	−9.804–20.770

Abbreviations: AUC_0–∞_, the area under the curve (0–∞); AUC_0–*t*_, the area under the curve (0–*t*); CI, confidence interval; *C*_max_, maximum concentration; SE, standard error.

**TABLE 7:** Overview of TEAEs (safety set).

	180 mg/m^2^	220 mg/m^2^	Total
*N*	26	3	29
Any TEAE, *n* (%)	26 (100.0)	3 (100.0)	29 (100.0)
Any TRAE, *n* (%)	26 (100.0)	3 (100.0)	29 (100.0)
SAE, *n* (%)	5 (19.2)	0 (0)	5 (17.2)
Any TEAE leading to a pause or stop of infusion, *n* (%)	5 (19.2)	1 (33.3)	6 (20.7)
Any TEAE leading to withdrawal, *n* (%)	3 (11.5)	0 (0)	3 (10.3)
Grade 3 or higher TEAEs, *n* (%)	9 (34.6)	2 (66.7)	11 (37.9)
Grade 3 or higher TEAEs of more than 5%, *n* (%)			
Diarrhea	3 (11.5)	0 (0)	3 (10.3)
Hypokalemia	2 (7.7)	1 (33.3)	3 (10.3)
Leukopenia	2 (7.7)	1 (33.3)	3 (10.3)
Neutropenia	2 (7.7)	1 (33.3)	3 (10.3)

Abbreviations: SAE, serious adverse event; TEAE, Treatment‐Emergent Adverse Event; TRAE, treatment‐related adverse event;

**TABLE 8:** Outcomes of antitumor activity (FAS).

	180 mg/m^2^	220 mg/m^2^	Total
*N*	26	3	29
CR	0	0	0
PR	10	0	10
SD	9	1	10
PD	5	1	6
NE	2	1	3
ORR			
*n* (%)	10 (38.5)	0 (0)	10 (34.5)
95% CI	20.2–59.4	–	17.9–54.3
DCR			
*n* (%)	19 (73.1)	1 (33.3)	20 (69.0)
95% CI	52.2–88.4	0.8–90.6	49.2–84.7

Abbreviations: CI, confidence interval; CR, complete response; DCR, disease control rate; FAS, full analysis set; NE, not evaluable; ORR, overall response rate; PD, progressive disease; PR, partial response; SD, stable disease.

**TABLE 9:** PFS and OS outcomes (EAS).

	180 mg/m^2^	220 mg/m^2^	Total
*N*	24	2	26
PFS (months)			
Events, *n* (%)	9 (37.5)	2 (100.0)	11 (42.3)
Censored, *n* (%)	15 (62.5)	0 (0)	15 (57.7)
50% (95% CI)	3.4 (2.7 to NA)	2.2 (1.6 to NA)	3.4 (2.4 to NA)
OS (months)			
Events, *n* (%)	11 (45.8)	1 (50.0)	12 (46.2)
Censored, *n* (%)	13 (54.2)	1 (50.0)	14 (53.8)
50% (95% CI)	12.1 (5.9 to NA)	NA (1.9 to NA)	12.1 (5.9 to NA)

Abbreviations: EAS, efficacy analysis set; OS, overall survival; PFS, progression‐free survival.

**TABLE 10:** PFS rate (%) in each month (EAS).

180 mg/m^2^ (*n* = 24)				220 mg/m^2^ (*n* = 2)			Total (*N* = 26)		
Time (months)	PD (*n*)	Number at risk (*n*)	PFS%	PD (*n*)	Number at risk (*n*)	PFS%	PD (*n*)	Number at risk (*n*)	PFS%
0	0	24	100	0	2	100	0	26	100
1	0	24	100	0	2	100	0	26	100
2	5	17	78.3	1	1	50	6	18	75.9
3	7	6	68.1	2	0	0	9	6	61.7
4	9	0	0				11	0	0

Abbreviations: EAS, efficacy analysis set; PD, progressive disease; PFS, progression‐free survival.

**TABLE 11:** OS rate (%) in each month (EAS).

180 mg/m^2^ (*n* = 24)				220 mg/m^2^ (*n* = 2)			Total (*N* = 26)		
Time (mon)	Death (*n*)	Number at risk (*n*)	OS%	Death (*n*)	Number at risk (*n*)	OS%	Death (*n*)	Number at risk (*n*)	OS%
0	0	24	100	0	2	100	0	26	100
1	0	24	100	0	2	100	0	26	100
2	1	23	95.8	1	1	50	2	24	92.3
3	1	23	95.8	1	1	50	2	24	92.3
4	4	20	83.3	1	1	50	5	21	80.8
5	6	18	75	1	1	50	7	19	73.1
6	7	17	70.8	1	1	50	8	18	69.2
7	7	17	70.8	1	1	50	8	18	69.2
8	7	16	70.8	1	1	50	8	17	69.2
9	9	14	62	1	1	50	10	15	61.1
10	10	11	56.8	1	1	50	11	12	56.4
11	10	9	56.8	1	1	50	11	10	56.4
12	10	4	56.8	1	1	50	11	5	56.4
13	11	2	42.6	1	0	50	12	2	45.1
14	11	2	42.6				12	2	45.1
15	11	1	42.6				12	1	45.1
16	11	0	42.6				12	0	45.1

Abbreviations: EAS, efficacy analysis set; OS, overall survival; PD, progressive disease;

### 3.3 Safety profile

All patients (100%) experienced at least one TEAE concerning JK1201I therapy. 11 (37.9%) experienced grade 3 or higher TEAEs, mostly diarrhea (*n* = 3, 10.3%), neutropenia (*n* = 3, 10.3%), leukopenia (*n* = 3, 10.3%), and hypokalemia (*n* = 3, 10.3%). In six patients (20.7%), the treatment was paused or stopped due to TEAE. TEAEs causing study withdrawal were in three patients (10.3%): one (3.4%) experienced rapid‐onset anaphylaxis^14^, ^15^; one died (3.4%) of pulmonary mucormycosis; and one experienced grade 4 diarrhea and neutropenia with a UGT1A1*6 (A/A) homozygosity mutation concerning severe irinotecan‐related neutropenia.^16^ These three cases were in the 180‐mg/m^2^ group. Two in the 180‐mg/m^2^ group experienced SAEs. One experienced grade 3 diarrhea, probably concerning JK1201I therapy, with diarrhea 7–8 times daily. The other experienced drug‐induced liver injury. Grade 3 or higher TEAEs were in over 5%, including diarrhea, 11.5% (3/26); neutropenia, 7.7% (2/26); leukopenia, 7.7% (2/26); and hypokalemia, 7.7% (2/26) in the 180‐mg/m^2^ group; and neutropenia, 33.3% (1/3) and leukopenia; 33.3% (1/3) in the 220‐mg/m^2^ group (Table 7).

### 3.4 Preliminary efficacy

**FIGURE 2:**
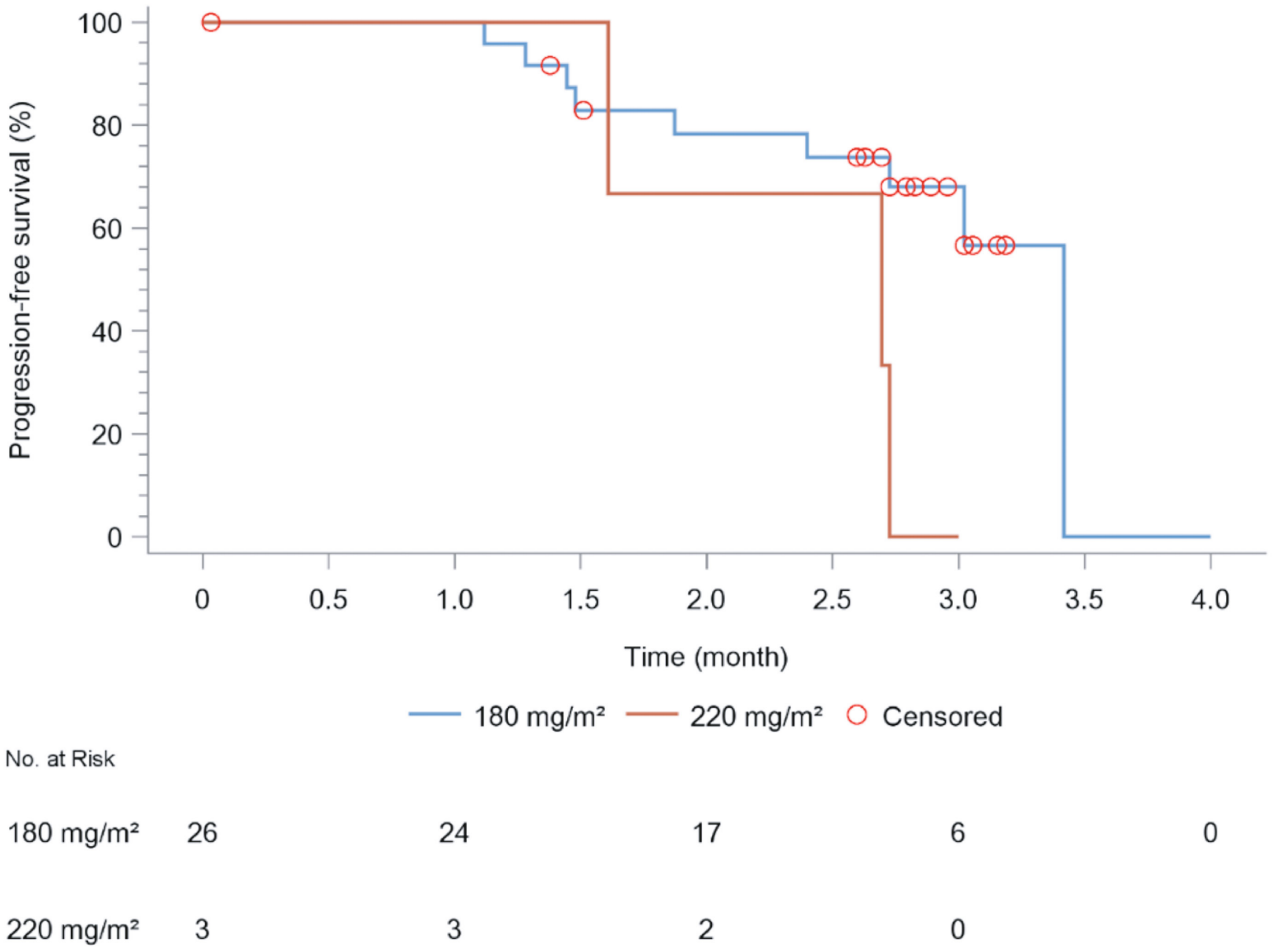
Estimated progression‐free survival (PFS) of patients under different dosages of JK1021I.

**FIGURE 3:**
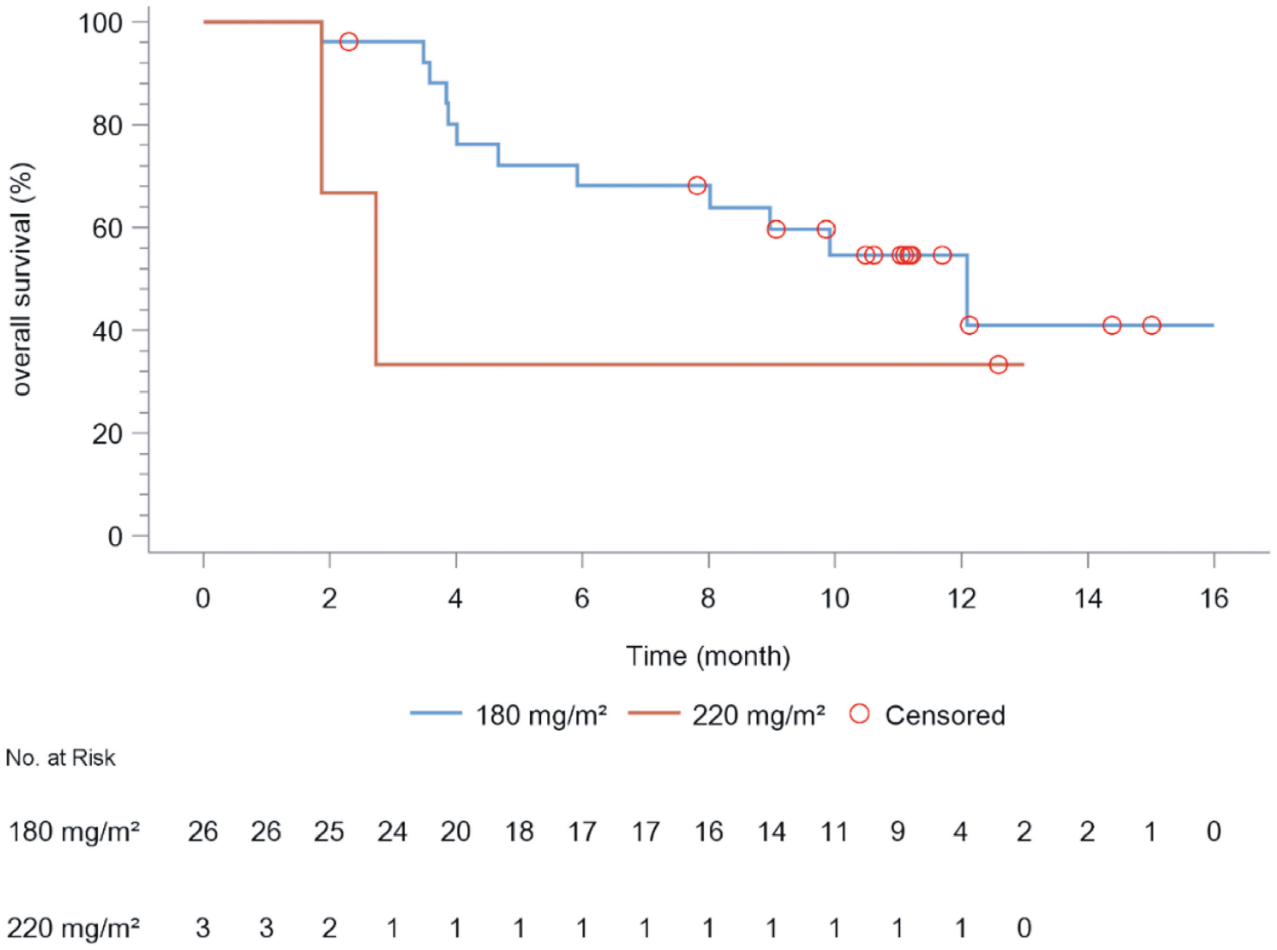
Estimated overall survival (PFS) of patients under different dosages of JK1021I.

Following the revised RECIST guideline (version 1.1), in the 180‐mg/m^2^ group, PR was achieved in 10 patients, stable disease was seen in nine, PD was in five, and two were not assessable. The ORR, DCR, mPFS, and mOS were 38.5% (10/26; 95% CI: 20.2–59.4), 73.1% (19/26; 95% CI: 52.2–88.4), 3.4 months (95% CI: 2.7 months to NA), and 12.1 months (95% CI: 5.9 months to NA). In the 220‐mg/m^2^ group, one each exhibited stable disease and PD; the remaining patient was not assessable. The ORR, DCR, and mPFS were 0% (0/3), 33.3% (1/3; 95% CI: 0.8–90.6), and 2.2 months (95% CI: 1.6 months to NA), respectively, while mOS could not be determined (Tables 8 and 9; Figures 2 and 3). Data collection for PFS concluded posttreatment, causing a PFS rate of 0% in the fourth month (Table 10). OS data were updated every 3 months until the electronic data capture system closed on April 11, 2023, with a 16‐month OS rate of 42.6% in the 180‐mg/m^2^ group (Table 11). Figures 2 and Figure 3 depict KM estimates for PFS and OS.

**FIGURE 4:**
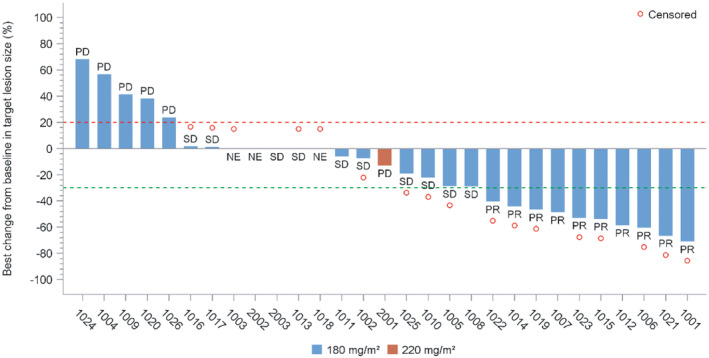
Best change in the target lesion size from baseline (full analysis set).

**FIGURE 5:**
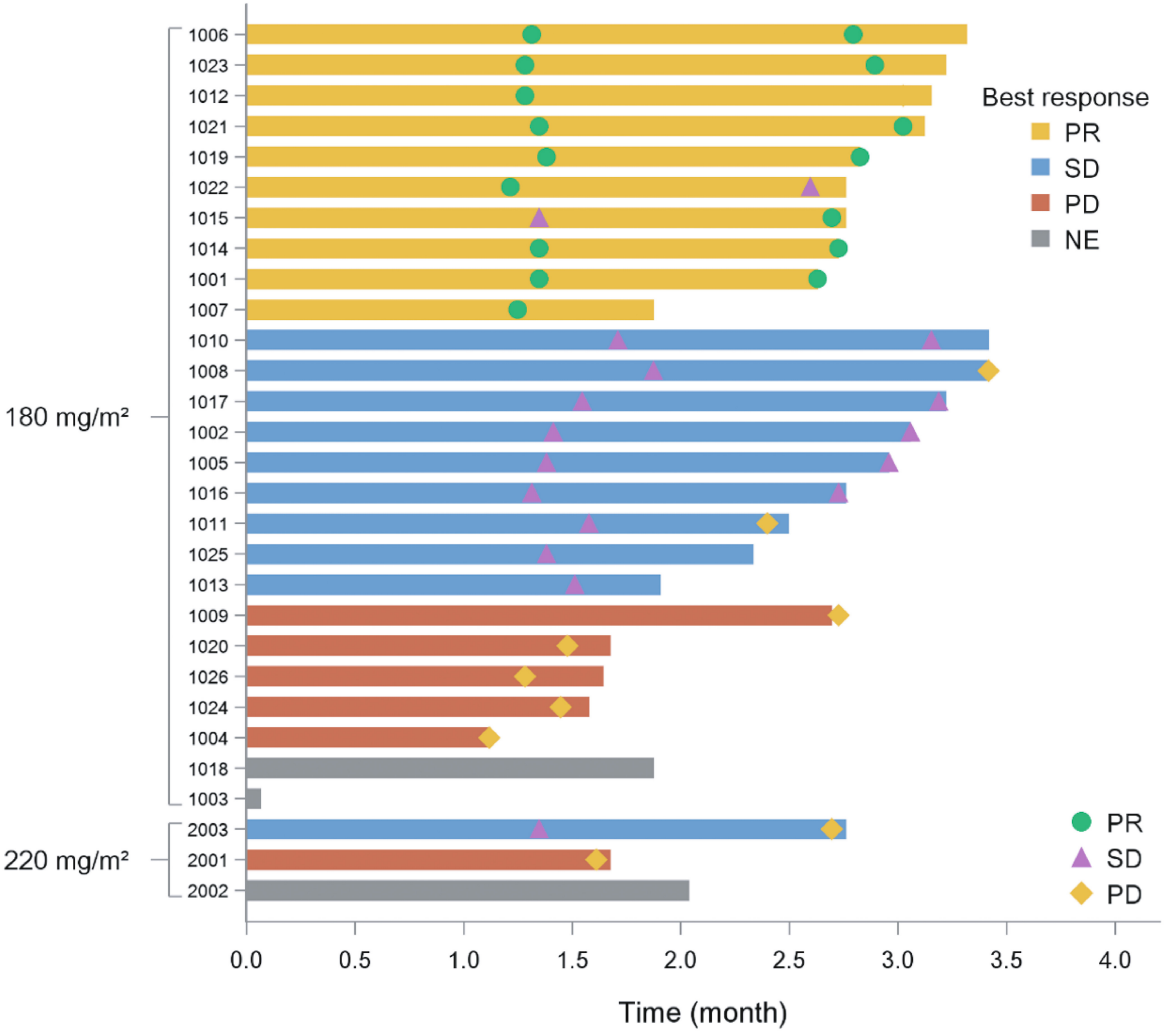
Waterfall plot describing best response and corresponding duration.

Post hoc supplementary analysis results in the 180‐mg/m^2^ group revealed that mOS was longer in patients under 65 years than those over 65 (12.1 vs. 5 months). The maximum OS was 15.01 months in a 45‐year‐old patient (enrolled as #1006). This individual presented with brain and lymphatic metastases but no bone metastases and had an ECOG performance status of 0 at baseline. Immunotherapy was administered as the first‐line treatment. The period between the conclusion of the first‐line treatment and the initial administration of JK1201I was 6.6 months. The patient completed all four cycles of treatment, with a total exposure time of 67 days, marginally above the median of 63.5 days. The greatest reduction in target lesion size was recorded at −60.2% from the baseline (Figure 4), with the outcomes being PR (Figure 5). The shortest mOS, recorded at 3.58 months, was in a 65‐year‐old patient (#1004) who had lymphatic metastases but no brain or bone metastases and an ECOG score of 1 at baseline. This patient, receiving immunotherapy as the initial treatment, had 2 months between the end of the first‐line treatment and the first dose of JK1201I. Due to disease progression, only two treatment cycles were completed, with the target lesion size change of 56.33% (Figure 4). The mOS of patients experiencing vomiting and patients who did not were 9.9 and 12.1 months.

## 4 DISCUSSION

In our present study, JK1201I exhibited a 6‐month OS rate of 75%, a 1‐year OS rate of 56.8%, an ORR of 38.5%, and an mOS of 12.1 months in the 180‐mg/m^2^ group (Tables 8, 9, and 11). These results are also consistent with the meta‐analysis performed by Horita et al. involving 1347 patients across 14 articles,^17^ which reported 6‐month and 1‐year OS rates of 37% and 9% in refractory/relapsed patients, and 57% and 27% in sensitive/relapsed patients. Lurbinectedin is approved based on the 6‐month and 1‐year OS rates of 67.1% and 34.2%, an ORR of 35.2%, and an mOS of 9.3 months.^6^ Patients are not categorized into refractory/relapsed or sensitive/relapsed groups. Based on the dose selection results from the studies above, results suggest that 180 mg/m^2^ of JK1201I prolongs the OS of SCLC patients.

Horita et al.^17^ reported grade III/IV hematological AEs with topotecan treatment. The most common AEs reported with lurbinectedin therapy are neutropenia (46%), leucopenia (29%), thrombocytopenia (7%), and anemia (9%).^6^ Neutropenia (16%), abdominal sepsis (8%), thrombocytopenia (8%), and anemia (8%) are the most common AEs reported with liposomal irinotecan therapy.^7^ Kondo et al.^5^ administered 100 mg/m^2^ of irinotecan to patients with SCLC on days 1 and 8 every 3 weeks, reporting an OS of 10.4 months. The most common AEs were neutropenia (36.7%), thrombocytopenia (3.3%), anemia (13.3%), and febrile neutropenia (6.6%).^4^ Grade III/IV hematological AEs included neutropenia (7.7%), leucopenia (7.7%), hypokalemia (7.7%), thrombocytopenia (3.8%), and anemia (3.8%). Grade III/IV gastrointestinal AEs included diarrhea (11.5%) in the 180‐mg/m^2^ group. Lower toxicity rates are observed with JK1201I therapy. Findings were reported by SW Song et al. Based on the histological examination of bone marrow and thymus tissue, no significant difference was observed between the toxicity rates of 80 mg/kg of JK1201I and normal saline. However, a decreasing number of hematopoietic stem cells in the bone marrow and lymphocytes in the thymus was observed in rats administered 40 mg/kg of irinotecan.^11^

UGT1A1 homozygosity mutation is a high‐risk SAE effector of irinotecan and SN38. The US FDA recommends reducing the initial dose of irinotecan in patients with UGT1A1*28 homozygous mutations.^18^ Nevertheless, compared with UGT1A1*28 (TA 7/7) mutations, UGT1A1*6 (A/A) mutations are more common in Japanese patients (5.7%–6.3%).^19^, ^20^ Bai et al.^21^ reported that 2.47% of UGT1A1*6 (A/A) mutations and 4.94% of UGT1A1*28 (TA 7/7) mutations were found in Chinese patients with cancer. No grade III/IV diarrhea and neutropenia were found in the phase I study; the genetic examination of UGT1A1 was not planned. Patient #1013 experienced grade 4 diarrhea and neutropenia associated with JK1201I treatment. A post hoc genetic examination revealed the presence of UGT1A1*6 (A/A) homozygosity mutation. Therefore, the genetic examination of UGT1A1 must be performed in subsequent studies on pegylated irinotecan.

## 5 CONCLUSION

The intravenous administration of 180 mg/m^2^ of JK1201I once every 3 weeks as a second‐line monotherapy exhibited low toxicities. These findings can be further verified by performing a phase III trial comparing JK1201I with topotecan, which we intend to conduct in 2024.

## AUTHOR CONTRIBUTIONS

**Jieran Long:** Resources (equal). **Xuefei Li:** Writing – original draft (equal). **Lin Wu:** Resources (equal). **Guohua Yu:** Resources (equal). **Aimin Zang:** Resources (equal). **Yanqiu Zhao:** Resources (equal). **Jinsheng Shi:** Resources (equal). **Ligong Nie:** Resources (equal). **Xuan Zhao:** Resources (equal). **Jian Fang:** Resources (equal).

## FUNDING INFORMATION

This work was supported by the JenKem Technology Co. Ltd. (Tian Jin).

## CONFLICT OF INTEREST STATEMENT

The authors declare no conflicts of interest.

## ETHICS STATEMENT

The study was approved by the ethics review board of Beijing University Cancer Hospital (No. 2021YW162) in accordance with the Declaration of Helsinki. All methods were carried out in accordance with relevant guidelines and regulations.

## PATIENT CONSENT STATEMENT

Written informed consent was obtained from all individual patients included in the study.

## Data Availability

The original contributions presented in the study are included in the article/Supplementary Material. Further inquiries can be directed to the corresponding authors.

## References

[1] Zhong YJ , Wen YF , Wong HM , Yin G , Lin R , Yang SY . Trends and patterns of disparities in burden of lung cancer in the United States, 1974–2015. Front Oncol. 2019;9:404. 31214489 10.3389/fonc.2019.00404 PMC6555199

[2] Zhang R , Li P , Li Q , et al. Radiotherapy improves the survival of patients with extensive‐disease small‐cell lung cancer: a propensity score matched analysis of surveillance, epidemiology, and end results database. Cancer Manag Res. 2018;10:6525‐6535. 30555258 10.2147/CMAR.S174801 PMC6278721

[3] Ardizzoni A , Hansen H , Dombernowsky P , et al. Topotecan, a new active drug in the second‐line treatment of small‐cell lung cancer: a phase II study in patients with refractory and sensitive disease. The European Organization for Research and Treatment of cancer early clinical studies group and new drug development office, and the lung cancer cooperative group. J Clin Oncol. 1997;15(5):2090‐2096. 9164222 10.1200/JCO.1997.15.5.2090

[4] O'Brien ME , Ciuleanu TE , Tsekov H , et al. Phase III trial comparing supportive care alone with supportive care with oral topotecan in patients with relapsed small‐cell lung cancer. J Clin Oncol. 2006;24(34):5441‐5447. 17135646 10.1200/JCO.2006.06.5821

[5] Kondo R , Watanabe S , Shoji S , et al. A phase II study of Irinotecan for patients with previously treated small‐cell lung cancer. Oncology. 2018;94(4):223‐232. 29444512 10.1159/000486622

[6] Trigo J , Subbiah V , Besse B , et al. Lurbinectedin as second‐line treatment for patients with small‐cell lung cancer: a single‐arm, open‐label, phase 2 basket trial. Lancet Oncol. 2020;21(5):645‐654. 32224306 10.1016/S1470-2045(20)30068-1

[7] Paz‐Ares L , Spigel DR , Chen Y , et al. RESILIENT part 1: a phase 2 dose‐exploration and dose‐expansion study of second‐line liposomal irinotecan in adults with small‐cell lung cancer. Cancer. 2022;128(9):1801‐1811. 35195913 10.1002/cncr.34123

[8] IPSEN . Ipsen announces results from Phase III RESILIENT trial evaluating Onivyde® in second‐line monotherapy for small‐cell lung cancer 2022. Available from: https://www.ipsen.com/press‐releases/ipsen‐announces‐results‐from‐phase‐iii‐resilient‐trial‐evaluating‐onivyde‐in‐second‐line‐monotherapy‐for‐small‐cell‐lung‐cancer/

[9] Rudin CM , Dowlati A , Chen Y , et al. 161O RESILIENT part 2: a randomized, open‐label phase III study of liposomal irinotecan versus topotecan in adults with relapsed small‐cell lung cancer (SCLC). J Thorac Oncol. 2023;18(4):S129‐S130. 10.1200/JCO.23.02110 PMC11210946 38648575

[10] Xu L , Huang W , Zhao X . Polyethylene glycol‐amino acid oligopeptide‐irinotecan drug conjugated and drug composition there of China. Beijing Arete Intellectual Property Agency; 2013.

[11] Song S , Sun D , Wang H , et al. Full‐profile pharmacokinetics, anticancer activity and toxicity of an extended release trivalent PEGylated irinotecan prodrug. Acta Pharm Sin B. 2023;13(8):3444‐3453. 37655324 10.1016/j.apsb.2023.01.011 PMC10466002

[12] Eisenhauer EA , Therasse P , Bogaerts J , et al. New response evaluation criteria in solid tumours: revised recist guideline (Version 1.1). Eur J Cancer. 2009;45(2):228‐247. 19097774 10.1016/j.ejca.2008.10.026

[13] Pitot HC , Goldberg RM , Reid JM , et al. Phase I dose‐finding and pharmacokinetic trial of irinotecan hydrochloride (CPT‐11) using a once‐every‐three‐week dosing schedule for patients with advanced solid tumor malignancy. Clin Cancer Res. 2000;6(6):2236‐2244. 10873073

[14] El‐Haik Y , Vitte J , Gasmi M , Birnbaum J , Default A , Jean‐Pastor M . Anaphylactic reaction during infusion of irinotecan. Fundam Clin Pharmacol. 2014;28:53.23025717

[15] Abu‐Amna M , Hassoun G , Hadad S , Haim N , Bar‐Sela G . Successful desensitization protocol for hypersensitivity reaction caused by Irinotecan in a patient with metastatic colorectal cancer. Clin Colorectal Cancer. 2015;14(4):e49‐e51. 26051444 10.1016/j.clcc.2015.05.003

[16] Xu JM , Wang Y , Ge FJ , Lin L , Liu ZY , Sharma MR . Severe irinotecan‐induced toxicity in a patient with UGT1A1 28 and UGT1A1 6 polymorphisms. World J Gastroenterol. 2013;19(24):3899‐3903. 23840132 10.3748/wjg.v19.i24.3899 PMC3699040

[17] Horita N , Yamamoto M , Sato T , et al. Topotecan for relapsed small‐cell lung cancer: systematic review and meta‐analysis of 1347 patients. Sci Rep. 2015;5:15437. 26486755 10.1038/srep15437 PMC4614251

[18] Pfizer Inc . Package Insert of Camptosar® (Irinotecan Hydrochloride) Injection: Pfizer Medical Information. 2022. Available from: https://www.pfizermedicalinformation.com/camptosar/highlights

[19] Akiyama Y , Fujita K , Nagashima F , et al. Genetic testing for UGT1A1*28 and *6 in Japanese patients who receive irinotecan chemotherapy. Ann Oncol. 2008;19(12):2089‐2090. 18953066 10.1093/annonc/mdn645 PMC2733123

[20] Fujii H , Yamada Y , Watanabe D , et al. Dose adjustment of irinotecan based on UGT1A1 polymorphisms in patients with colorectal cancer. Cancer Chemother Pharmacol. 2019;83(1):123‐129. 30377777 10.1007/s00280-018-3711-8 PMC6373181

[21] Bai Y , Wu HW , Ma X , Liu Y , Zhang YH . Relationship between UGT1A1*6/*28 gene polymorphisms and the efficacy and toxicity of irinotecan‐based chemotherapy. Onco Targets Ther. 2017;10:3071‐3081. 28790841 10.2147/OTT.S137644 PMC5488790

